# Lifewide profile of cytokine production by innate and adaptive immune cells from Brazilian individuals

**DOI:** 10.1186/s12979-017-0084-5

**Published:** 2017-01-23

**Authors:** Gabriela Silveira-Nunes, Elaine Speziali, Andréa Teixeira-Carvalho, Danielle M. Vitelli-Avelar, Renato Sathler-Avelar, Taciana Figueiredo-Soares, Maria Luiza Silva, Vanessa Peruhype-Magalhães, Daniel Gonçalves Chaves, Gustavo Eustáquio Brito-Melo, Glenda Meira Cardoso, Eric Bassetti Soares, Silvana Maria Elói-Santos, Rosângela Teixeira, Dulciene Magalhães Queiroz, Rodrigo Corrêa-Oliveira, Ana Maria Caetano Faria, Olindo Assis Martins-Filho

**Affiliations:** 1 Departamento de Bioquímica e Imunologia, Instituto de Ciências Biológicas, Universidade Federal de Minas Gerais, Avenida Antônio Carlos 6627, Belo Horizonte, Minas Gerais 31270-901 Brazil; 2 Laboratório de Biomarcadores de Diagnóstico e Monitoração, Centro de Pesquisas René Rachou, FIOCRUZ, Belo Horizonte, Brazil; 3 Faculdade de Medicina, Universidade Federal de Minas Gerais, Belo Horizonte, Brazil; 4 Laboratório de Imunologia Celular e Molecular, Centro de Pesquisas René Rachou, FIOCRUZ, Belo Horizonte, Brazil; 5 Laboratório de Pesquisas Clínicas, Centro de Pesquisas René Rachou, FIOCRUZ, Belo Horizonte, Brazil; 6 Departamento de Farmácia, Universidade Federal dos Vales do Jequitinhonha e Mucuri, Diamantina, Minas Gerais Brazil; 7 Maternidade Odete Valadares/Fundação Hospitalar do Estado de Minas Gerais (FHEMIG), Belo Horizonte, Brazil

**Keywords:** Aging, Cytokine, Adaptive immune cells, Innate immune cells

## Abstract

**Background:**

Immunosenescence is associated with several changes in adaptive and innate immune cells. Altered cytokine production is among the most prominent of these changes. The impact of age-related alterations on cytokine global profiles produced by distinct populations of leukocytes from healthy Brazilian individuals was studied. We analysed frequencies of cytokine-producing lymphocytes and innate immune cells from individuals at several ages spanning a lifetime period (0–85 years).

**Results:**

Healthy adult individuals presented a balanced profile suggestive of a mature immune system with equal contributions of both innate and adaptive immunity and of both categories of cytokines (inflammatory and regulatory). In healthy newborns and elderly, innate immune cells, especially neutrophils and NK-cells, contributed the most to a balanced profile of cytokines.

**Conclusions:**

Our results support the hypothesis that ageing is not associated with a progressive pro-inflammatory cytokine production by all leukocytes but rather with distinct fluctuations in the frequency of cytokine-producing cells throughout life.

**Electronic supplementary material:**

The online version of this article (doi:10.1186/s12979-017-0084-5) contains supplementary material, which is available to authorized users.

## Background

In the past decade, a new approach to the study of ageing emerged from the data collected from centenarians. The concept of successful or healthy ageing comes from these studies and it eliminated the confusion between ageing and age-related disorders. According to these studies, immunosenescence does not involve a simple unidirectional decline in all functions, but rather a remodeling of biological systems during the ageing process. In this sense, many immunological activities are well preserved in the healthy elderly and they may compensate for other functions that are impaired [1–3].

Ageing is associated with several alterations in the phenotype, repertoire and activation status of leukocytes as well as in the cytokine profile produced by these cells. This complex age-related remodeling of the immune system is responsible for the profound changes within the cytokine network [3–8]. Cytokines are a key component in the communication among immune cells and they are responsible for differentiation, proliferation and survival of lymphoid cells, playing an important role in immune responses and inflammation. Age-related changes in the cytokine network is responsible for a chronic proinflammatory status, known as “inflammageing” [3, 5]. Inflammageing has been described as a combination of dysfunctional immunity with a state of low grade chronic inflammation and it has been considered as an universal phenomenon associated with frailty and morbidity in the elderly [3, 8–11]. This progressive increase in pro-inflammatory status is one of the major characteristics of immunosenescence [12–14].

Several ageing-associated immunological alterations have been already described in medical literature, mostly in the T-cell compartment. They include involution of the thymus, reduction in the number of naïve T-cells with a parallel increase of oligoclonaly expanded CD4+ T-cells with a memory phenotype, reduced potential to produce IL-2 and loss of CD28 expression [15–17]. Adaptive immunity undergoes severe deterioration with age and this represents the main problem in the elderly. However, evidence accumulated over the last decade supports the hypothesis that ageing has also a profound impact on innate immunity, which in turn markedly influences health and longevity of older people [3, 18–20].

In the complex scenario of immunosenescence, it has been generally accepted that some aspects of innate immunity, e.g., phagocytosis and natural killer (NK)-cell cytotoxicity, remain largely unaffected [19–21]. Innate immune responses are more resistant to change and NK-cells are well preserved in healthy elderly subjects. In fact, there is an age-related increase in CD16+ CD57- cells with high cytotoxicity capacity. This increase in NK cells has been correlated with successful ageing [21–24]. Our group reported a significant increase in frequency of CD16+ IFN-γ+ NK-cells in aged individuals in schistosomiasis endemic areas of Brazil who were protected from schistosome infection. Therefore, a high frequency of IFN-γ+ NK-cells correlated with “healthy ageing” in endemic areas [22].

Studies in aged mice showed functional decline of monocytes and macrophages, low expression level of Toll-like receptors from activated splenic and peritoneal macrophages and altered secretion of several chemokines and cytokines [25, 26]. Reduced class II major histocompatibility in aged macrophages also contribute to impaired proliferative response of activated peripheral T-lymphocytes [20, 27–29].

In humans, it has been described that although the elderly preserve the number and phagocytic capacity of neutrophils, other functional characteristics of these cells such as superoxide anion production, chemotaxis, and apoptosis are reduced during ageing [18, 30, 31].

In this sense, healthy immunosenescence is the net result of a continuous adaptation of the body to deteriorative changes occurring over time. According to this concept, body resources are continuously optimized, and successful immunosenescence must be considered a very dynamic process of immunological remodeling [32, 33].

Most of the studies investigating the influence of inflammageing in the elderly were obtained at particular age intervals and most of them come from Caucasian individuals from either Europe or the United States. Even among these reports, authors have observed variations and contrasting results as their samples vary demographically and geographically [34–36]. The study of various age groups should yield more meaningful data on immunosenescence that could be directly used by the geriatricians to restore or attenuate deregulated immune responses and assure a healthy longevity.

In this study, we described age-related changes in the frequency of cytokine-producing leukocyte populations in order to contribute to the establishment of new reference parameters for Brazilian individuals.

## Methods

### Aim of the study

The aim was to establish new reference parameters of cytokine production by leukocytes for Brazilian healthy individuals at different ages. Since there are controversies on the topic of cytokine production during ageing due to differences in experimental designs [37], this study is part of a broad effort to identify cell subsets involved in the production of distinct patterns of cytokines during healthy ageing.

### Study population

The study population consisted of 181 healthy subjects: 35 children, 22 adolescents and 124 adults (age range 0–85 years). Blood samples were obtained from children visiting clinics for routine pediatric inspection. Adult samples were obtained from healthy individuals who accepted to participate in the study. Subjects were divided into 6 age categories: Newborn – 0 years (*n* = 12); Children – 6 – 10 years (*n* = 23); Adolescent – 11 – 20 years (*n* = 22); Adults – 21–50 (*n* = 80); Middle Aged – 51–60 (*n* = 22); Elderly – 61–85 (*n* = 22). These age ranges were set based on the main physiological changes occurring during lifetime (birth, childhood, adolescence, young and middle adulthood, senescence) as well as similar patterns of variation observed for cytokine-producing innate cells (Additional file 1: Figure S1) and lymphocytes (Additional file 2: Figure S2) throughout life. These volunteers were all residents in the state of Minas Gerais (Southeast Brazil).

All individuals were interviewed by a health practitioner using a health questionnaire, submitted to a physical exam and to hematological and biochemical tests to establish their health condition. The exclusion criteria for both populations were: infections, acute or chronic inflammation, autoimmune diseases, heart disease, undernourishment, anemia, leucopoenia, mood disorders, neurodegenerative disease, neoplasias and use of hormones (steroids) and drugs (alcohol, antidepressants, immunosuppressants, anticoagulants). Children were excluded of the study if they showed any evidence of congenital disease, infection, immunological disorder or were taking any concomitant medications. Written informed consent forms were obtained from each participant, their parents or guardians prior to their inclusion in our study. This work was approved by the Ethical Committees of FIOCRUZ (Ministry of Health, Belo Horizonte, Brazil) as well as the National Research Ethics Committees (CONEP) of Brazil.

### Blood collection

Nine milliliters (9 mL) of blood were drawn from each individual always in the morning period to avoid circadian variations.

### Short-term whole blood culture and intracytoplasmic cytokine staining

Aliquots of 500 μL peripheral blood were placed in 5 mL polypropylene tubes containing 500 μl RPMI-1640 added and incubated for six hours at 37 ºC and 5% CO_2_. Next, 10 μL Brefeldin A (BFA; Sigma Chemical Company, St. Louis, MO) (10 mg/mL final concentration) was added and incubated for four hours at 37 ºC and 5% CO_2_. Then, 110 μL ethylenediamine tetraacetic acid 20 mM (EDTA; Sigma Chemical Company), (final concentration of 2 mM) solution was added. Tubes were incubated for 15 min at room temperature. Next, 4 mL PBS-W (cell wash solution) was added and samples were centrifuged for 10 min at 400 x g and 18 ºC. Supernatants were collected and aliquots stained with fluorescent-labeled anti-human cell surface monoclonal antibodies (to CD4, CD8, CD14, CD16 and CD19) for 30 min at room temperature and protected from light. After membrane staining, erythrocyte lysis, and leukocytes fixation, cell suspensions were permeabilized with PBS-P (PBS 0.05M pH 7.4 containing 0.5% BSA, 0.1% sodium azide and 0.5% saponin: SIGMA, St. Louis, MO, USA) and aliquots incubated for 30 min at room temperature, in the dark, with fluorescent-labeled anti-cytokine monoclonal antibodies to IL-4, IL-5, IL-10, TNF-α and IFN-γ (BD-Pharmingen, San Jose, CA). After intracytoplasmic cytokine staining, leucocytes were washed with PBS-W and fixed in fixative solution Macs Facs Fix (MFF). Samples were analyzed by flow cytometry for the production of TNF-α, IFN-γ, IL-4, IL-5 and IL-10 by CD4+ T-cells and CD8+ T-cells, and the production of TNF-α, IL-4 and IL-10 by CD19+ B cells. Additionally, production of TNF-α, IFN-γ, IL-4 and IL-10 by neutrophils, production of TNF-α and IL-10 by monocytes and production of TNF-α, IFN-γ and IL-4 by NK-cells were analyzed.

### Flow cytometry acquisition and analysis

Flow cytometry acquisition and analysis were performed in a FACScalibur™ equipped with a four-color detection system (Becton Dickinson, San Jose, CA, USA), using the CELLQUEST software (Franklin Lakes, NJ, USA). After acquiring 30,000 events/tube, distinct gating strategies were used to analyze the different cytokine-expressing leukocytes subsets, including innate immune cells (neutrophils, monocytes and NK-cells) and adaptive immune cells (CD4+, CD8+ T-cell subsets and B-cells). Selective analysis of neutrophils was performed by establishing a specific scatter gate using the dot plot distribution of anti-CD16-FITC and laser side scatter (SSC) to discriminate the neutrophils as SSC high CD16high+. Analysis of monocytes was performed using the dot plot distribution of anti-CD14-TC and SSC to discriminate the monocytes as SSC int CD14high+ cells. Selection of NK-cells, T-cell subsets and B-cells was performed by gating lymphocytes on forward scatter (FSC) versus SSC dot plot distribution, followed by analysis based on anti-CD16-FITC, anti-CD19-FITC, anti-CD8-FITC or anti-CD4-TC labelling. Following the selection of leucocyte subsets, frequency of cytokine positive cells was determined using quadrant statistics over FL-1/anti-cell surface marker-FITC or FL-3/anti-cell surface marker-TC versus FL-2/anti-cytokine-PE dot plot distribution. Results were expressed as percentages of cytokine positive cells for different gated leucocyte subpopulations analyzed.

### Cytokine signature analysis

Cytokine-mediated immune response elicited by leukocytes in each age group was assessed after short-term in vitro culture with no stimulation. Analysis of the intracytoplasmic cytokine profile of peripheral blood leukocytes initially yielded the percentage of cytokine positive cells. Cytokine profiles produced by leukocytes were assessed to identify low (≤ global median) and high (>global median) frequencies of cytokine-producing cells using the global median of each cytokine as a cut-off. For the calculation of the global median the whole universe of data obtained for the groups was considered. Leukocyte subpopulations assessed included neutrophils (NEU), monocytes (MON), NK-cells as well as lymphocyte subsets and the global median of each sub-population was described in Table 1. This strategy allows multiple comparative analyzes among groups, neither of which is excluded from the analysis. The high frequency of cells producing cytokines was used to evaluate the overall cytokine patterns from healthy individuals categorized by age ranges.

**Table 1:** Global median of the universe of data on cytokine-producing leukocytes

Immune Compartment	Cytokine-producing leukocytes	Median (%)
Innate	TNF-α+ Neutrophils	0.51
	IFN-γ+ Neutrophils	0.34
	IL-4+ Neutrophils	0.43
	IL-10+ Neutrophils	0.19
	TNF-α+ Monocytes	32.20
	IL-10+ Monocytes	3.00
	TNF-α+ NK-cells	0.18
	IFN-γ+ NK-cells	0.21
	IL-4+ NK-cells	0.19
Acquired	TNF-α+ CD4+ T-cells	0.28
	IFN-γ+ CD4+ T-cells	0.26
	IL-4+ CD4+ T-cells	0.32
	IL-5+ CD4+ T-cells	0.11
	IL-10+ CD4+ T-cells	0.75
	TNF-α+ CD8+ T-cells	0.34
	IFN-γ+ CD8+ T-cells	0.28
	IL-4+ CD8+ T-cells	0.27
	IL-5+ CD8+ T-cells	0.14
	IL-10+ CD8+ T-cells	0.40
	TNF-α+ B-cells	0.41
	IL-4+ B-cells	0.30
	IL-10+ B-cells	0.57

Silveira-Nunes et al.

#### Comparative data analysis

**Fig. 1:**
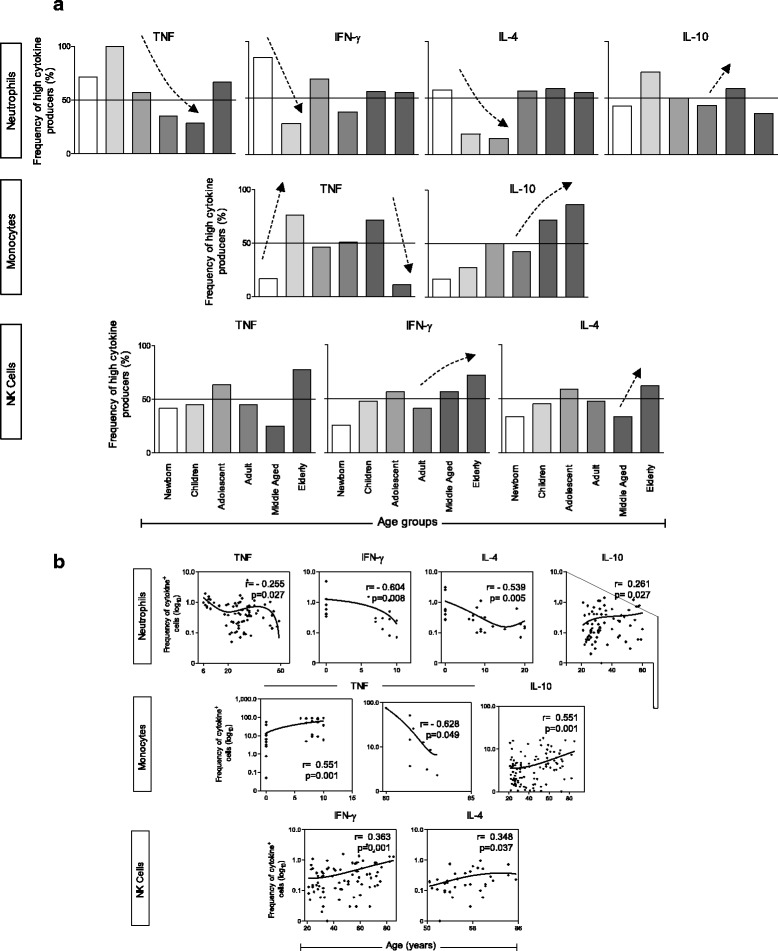
Profile of subjects with high frequency of neutrophils, monocytes and NK-cells producing proinflammatory and regulatory cytokines. **a** The cytokine profile is presented as percentage of individuals with high frequency of cytokine-producing cells in each age group: Newborn – 0 years (*n* = 12); Children – 6–10 years (*n* = 23); Adolescent – 11–20 years (*n* = 22); Adults – 21–50 (*n* = 80); Middle Aged – 51–60 (*n* = 22); Elderly – 61–85 (*n* = 22). Bars represent the percentage of subjects with high frequency of neutrophils producing tumor necrosis factor (TNF)-α, interferon (IFN)-γ, interleukin (IL)-4 and IL-10, monocytes producing TNF-α and IL-10, and NK-cells producing TNF-α, IFN-γ and IL-4. Relevant differences were considered when the percentage of individuals with high frequency cells producing a given cytokine emerged above the 50th percentile (*continuous line*). Spearman correlation was performed to evaluate either increase or decrease in frequency of cytokine-producing cells among age groups. Significant positive or negative correlations were represented by *dotted arrows* (↑). **b** Spearman’s rank correlation. Corresponding Pearson’s correlation coefficient (r) and *p* value between cytokine-producing cell frequency and age in years is shown

**Fig. 2:**
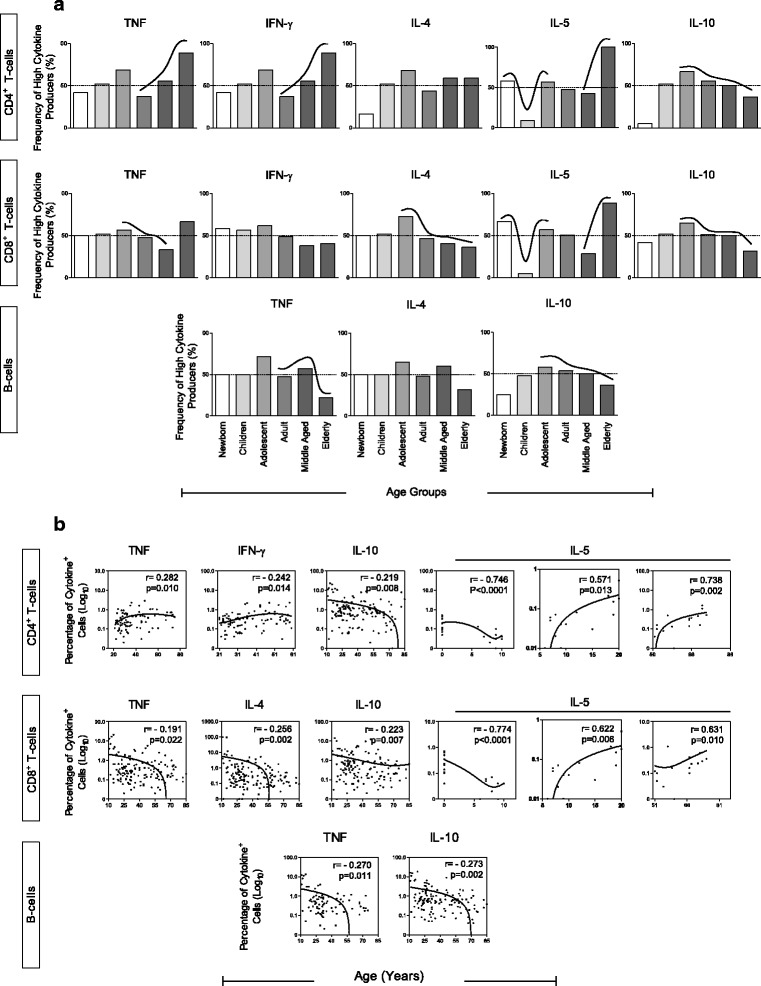
Profile of subjects with high frequency of CD4+ T-cells, CD8+ T-cells and B-cells producing proinflammatory and regulatory cytokines. **a** Cytokine profile is presented as percentage of individuals with high frequency of cytokine-producing cells in each age group: Newborn – 0 years (*n* = 12); Children – 6–10 years (*n* = 23); Adolescent – 11–20 years (*n* = 22); Adults – 21–50 (*n* = 80); Middle Aged – 51–60 (*n* = 22); Elderly – 61–85 (*n* = 22). Bars represents the percentage of subjects with high frequency of CD4 T-cells producing tumor necrosis factor (TNF)-α, interferon (IFN)-γ, interleukin (IL)-4, IL-5 and IL-10. CD8 T-cells producing TNF-α, IFN-γ, IL-4, IL-5 and IL-10. B-cells producing TNF-α, IFN-γ, IL-4, IL-5 and IL-10. Relevant differences were considered when the percentage of individuals with high frequency of cells producing a given cytokine emerged above the 50th percentile (*continuous line*). Spearman correlation was performed to evaluate either increase or decrease in frequency of cytokine-producing cells among age groups. Significant positive or negative correlations were represented by *dotted arrows* (↑). **b** Spearman’s rank correlation. Corresponding Pearson’s correlation coefficient (r) and *p* value between cytokine-producing cell frequency and age in years is shown

The type of analysis that we have used in the present investigation is not a conventional statistical analysis. This novel concept of cytokine signature has been proposed by Luiza-Silva and colleagues [38]. It is a new strategy to evaluate the overall cytokine profile instead of analyzing single cytokine profiles. To reinforce our data we also used the Spearman correlation test in our relevant findings. The immune profile is represented as percentage of individuals with high frequency of cytokine-producing innate and acquired immune cells. Relevant differences were considered when the percentage of individuals with a high frequency of cells producing a given cytokine emerged above the 50th percentile (Figs. 1 and 2). This approach showed to be acurate to detect subtle changes in cytokine signatures not detectable by conventional statistical approaches. Then, considering the relevant production of cytokines, Spearman correlation was performed to evaluate increase or decrease in frequency of cytokine-producing cells among age groups.

Since lymphocytes are known to be at higher numbers in newborn and children than in adults, the absolute numbers of lymphocytes for each age group is provided in Table 2. In spite of the variation in the number of these cells, cell frequencies are reliable parameters to evaluate the relative contribution of each cytokine-producing cells within a population.

**Table 2:** Number of lymphocytes in peripheral blood of individuals from each age group

Age groups	Total lymphocytes (mean + SD)^a^
Newborn	2848 + 1725
Children	2984 + 1310
Adolescent	2180 + 678
Adult	2270 + 753
Middle aged	2553 + 658
Elderly	1324 + 515

^a^Numbers represent the total lymphocytes + standard deviation for each age group. Numbers of lymphocytes were obtained by blood count using an Abbott Cell-Dyn 1700 automatic analyzer

#### Radar charts

Radar charts were used to summarize the proinflammatory (▄) versus regulatory () cytokine signatures in a range of leukocyte subsets of innate and adaptive immunity for each age group. This analysis highlights the contribution of different leukocytes subsets for the global balance of cytokines. On radar charts, each axis represents the percentage (%) of volunteers showing high frequency of cytokine-producing cells. The values of each axis can be connected to form a central polygonal area that represents the proinflammatory versus regulatory global balance. Increase or decrease of the central polygonal area reflects either a higher or a lower contribution of proinflammatory versus regulatory profile for each age group.

#### Ascendant cytokine profile

The high cytokine producing-cell strategy was also used to create, for each age group, an ascendant profile that allows to analyze the hierarchical behavior of cytokine-producing immune cells throughout life. For this analysis, a functional cytokine categorization was used: Inflammatory cytokines – red bars (TNF-α and IFN-γ) and regulatory cytokines – blue bars (IL-4, IL-5 and IL-10). Only those that had more than 50% frequency of cytokine-producing cells in the group were considered as relevant.

## Results

To address the question of how ageing influences the global profile of cytokine production by acquired and innate immune cells, healthy individuals were categorized in carriers of either low or high frequency of cytokine-producing cells as previously described by Luiza-Silva and co-workers [38]. This allowed calculation of the percentage of individuals with high frequency of cytokine-producing cells for each age group. To assess a functional cytokine profile, we classified cytokines as proinflammatory (TNF-α and IFN-γ) and regulatory (IL-4, IL-5 and IL-10) according to its functional characteristics during the ageing process. We took into account for this classification that Th1 cytokines (TNF-α and IFN-γ) were mostly involved in chronic inflammatory disorders associated with ageing (such as cardiovascular, autoimmune and degenerative diseases) [39–41]. In addition, cytokines such as IL-4, IL-5.and IL-10 usually predominate in tolerogenic compartments such as gut mucosa and maternal-fetal interface [42–44].

### There are distinct variations in frequency of cytokine-producing leukocyte subsets throughout life

To overview the production of proinflammatory and regulatory cytokines throughout life, we performed an evaluation of the cytokine profile produced by leukocyte subsets from the innate (neutrophils, monocytes and NK-cells) and adaptive (CD4+ T-cells, CD8+ T-cells and B-cells) immune compartments. In this analysis, individuals were categorized as carriers of either low or high frequency of cytokine-producing cells, using the global median as a cut-off as previously described [38]. Therefore, it was possible to calculate the percentage of individuals with high frequency of cytokine-producing cells for each age group.

As mentioned in the methods section, establishment of age groups was performed according to the rhythmic variation of cytokine-producing cells from both innate and acquired immune compartments of peripheral blood of individuals throughout life (Additional file 1: Figure S1 and Additional file 2: Figure S2). Overall distribution of cytokine+ neutrophils, monocytes (CD14+) and NK-cells (CD16+) (Additional file 1: Figure S1) and of cytokine+ T-cells (CD4+ and CD8+) and B-cells (CD19+) (Additional file 2: Figure S2) were plotted as a function of age (ranging from 0 to 85). Age ranges were established based on the overall variation rhythm observed, considering the moving mean of all cytokine+ cell subsets (continuous lines). A similar strategy was used previously by our group in a study analyzing rhythmic variations in the frequency of different subsets of peripheral blood monocytes in individuals from 0 to 86 years of age. These age groups were shown to represent a homogenous profile suitable to identify immunologically meaningful periods in life [34].

Of note, in the innate cytokine profile, there was the significant decrease of TNF-α-producing neutrophils from childhood to adulthood when it reached a plateau. IFN-γ and IL-4 decreased early in life until childhood. On the other hand, IL-10-producing neutrophils increased during adulthood (adult and middle aged individuals). TNF-α-producing monocyte frequency increased from birth until childhood, then, maintained high during adolescence and adulthood decreasing in the elderly. Interestingly, monocytes maintained a low frequency of IL-10-producing cells during early life and shifted towards a progressive high production from adulthood to senescence. IFN-γ-producing NK-cells and IL-4-producing NK-cells increased in frequency from adult and middle-aged groups, respectively, until senescence (Fig. 1).

Frequency of TNF-α-producing CD4+ T-cells had an increase during the ageing process (significantly from adulthood to senescence), while TNF-α-producing CD8+ T-cells and B-cells decreased their frequencies. Regarding IFN-γ production, only CD4+ T cells changed: they increased from adulthood to middle age. It could be observed a significant decrease of IL-4-producing CD8+ T cells throughout life (specially from adolescent to elderly groups). An interesting pattern could be found in the profile of IL-5-producing T-cells. At birth, there was a high frequency of both IL-5-producing CD4+ T-cells and CD8+ T-cells; then, these frequencies decreased during childhood to increase again in adolescence, being maintained at approximately 50% of frequency of high cytokine-producing cells during adulthood. From the fiftieth decade of life to senescence, the frequency of IL-5-producing CD4+ and CD8+ T-cells increased further. A remarkable finding is that all IL-10-producing lymphocytes (T and B-cells) had their frequency decreased from adolescence to senescence (Fig. 2).

### Profiles of high frequencies of proinflammatory- and regulatory-cytokine-producing cells in innate and adaptive immune compartments vary throughout life

**Fig. 3:**
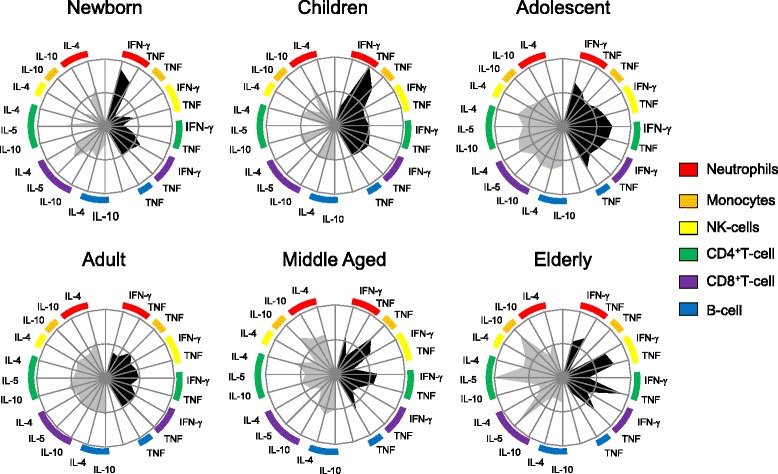
Radar graph representing the balance of subjects with high frequency of inflammatory (▄) or regulatory () cytokine-producing cells of innate and adaptive immunity. Graphs were constructed with each axis displaying the proportion of subjects with high frequency of cytokine-producing cells within a given leukocyte subset. The values of each axis can be joined to form the central polygon area that represents the general inflammatory/regulatory cytokine balance. Increasing or decreasing central polygon areas reflects either higher or lower contribution of inflammatory versus regulatory cytokine balance in each age group. Analysis of the radar chart axes highlights the contribution of distinct leukocyte subset for the overall cytokine balance. Age groups were categorized as: Newborn – 0 years (*n* = 12); Children – 6–10 years (*n* = 23); Adolescent – 11–20 years (*n* = 22); Adults – 21–50 (*n* = 80); Middle Aged – 51–60 (*n* = 22); Elderly – 61–85 (*n* = 22)

**Fig. 4:**
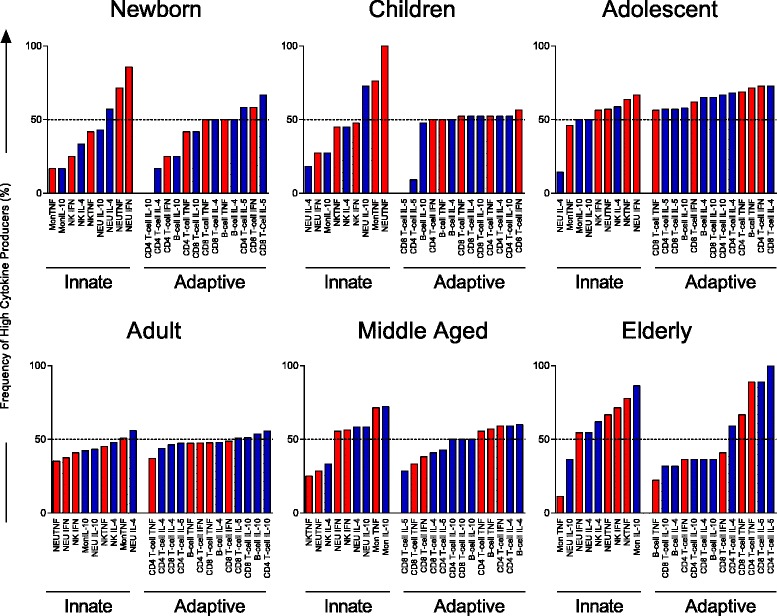
Signature of high frequency of cytokine producing-cells from innate and adaptive immune compartments by individuals of different age groups. Age groups were characterized as: Newborn – 0 years (*n* = 12); Children – 6–10 years (*n* = 23); Adolescent – 11–20 years (*n* = 22); Adults – 21–50 (*n* = 80); Middle Aged – 51–60 (*n* = 22); Elderly – 61–85 (*n* = 22). Ascendant frequency of cytokine-producing cells from innate and adaptive immune compartments in each group was represented by bars. Red bars represent inflammatory-cytokine-producing cells and the blue bars represent regulatory-cytokine-producing cells. Dotted line represent the 50^th^ percentile that was used as a cut-off to identify relevant differences

To further characterize the cytokine pattern of healthy individuals at distinct age groups, we have constructed radar charts for high frequency of cells producing proinflammatory and regulatory cytokines and an ascendant graph to characterize the hierarchical contribution of cytokine-producing cell types to the cytokine profile of each age group (Figs. 3 and 4).

Our data demonstrate that the cytokine pattern of newborns was characterized by a prominent participation of proinflammatory responses of the innate immune compartment (Fig. 3), especially driven by TNF-α and IFN-γ from neutrophils (Fig. 4). This profile seems to be counter-balanced by the presence of high frequencies of cells producing regulatory cytokines (mostly IL-5-producing T-cells) (Figs. 3 and 4).

According to our data, neutrophils had an important role in childhood. These innate cells, together with monocytes, produce large amounts of TNF-α, while neutrophils also produced high levels of IL-10, creating a compensatory circuit of cytokines (Figs. 3 and 4).

In the adolescent (11 to 20 years), we could observe a shift in the composition of the cytokine profile. In this age group, there was a predominance of adaptive immune responses, with participation of proinflammatory cytokines such as TNF-α from T and B-cells, and IFN-γ from CD4+ and CD8+ T-cells with a contribution of regulatory cytokines such as IL-4, IL-5 and IL-10 produced by T and B lymphocytes. Innate immune cells also influence the adolescent profile: TNF-α and IFN-γ produced by neutrophils and NK-cells, as well as IL-4 production by NK-cells (Figs. 3 and 4).

Interestingly, adults presented a profile well balanced between innate and adaptive immune responses with an equal contribution of proinflammatory and regulatory cytokine-producing cells (Figs. 3 and 4).

Another shift in the composition of the cytokine pattern could be observed from the fiftieth decade of life on. Middle aged group changed their profile from a balanced (seen in adulthood) to a higher contribution of the innate compartment of immune cells. Middle aged groups showed a high frequency of neutrophils producing TNF-α, IL-4 and IL-10, of monocytes producing TNF-α and IL-10, and of NK-cells producing IFN-γ. A prominent inflammatory profile of cytokines produced by lymphocytes was also observed in this age group (TNF produced by CD4+ T-cells and B-cells, IFN-γ produced by CD4+ T-cells and IL-4 produced by CD4 + T-cells and B-cells) (Figs. 3 and 4).

Changes in the cytokine pattern in the elderly group were characterized by a balanced profile with a prominent contribution of the innate immune response (driven by IL-10-producing monocytes, IL-4-producing neutrophils, TNF-α- and IFN-γ-producing neutrophils and NK-cells). Although innate immune cells are the most relevant element in the cytokine profile of this age group, the contribution of adaptive immune cells could still be identified as IL-5- and TNF-α-producing T-cells and IL-4-producing CD4+ T-cells.

## Discussion

Ageing influences the entire physiology of an organism, resulting in changes on functions at the molecular, cellular and systemic levels. Age-related physiological changes are well characterized in the immune system, which is continuously remodeled over the life course. One of these main remodeling changes during ageing occurs in the network of cytokine production [4, 8, 45].

In this study, we evaluated the profile of cytokine produced by major peripheral blood cells of individuals from various age groups. Interestingly, we observed that ageing was not associated with a progressive decline in cytokine production by all leukocyte subsets but it was rather characterized by distinct fluctuations of cytokines produced at various time points during a lifetime period. Furthermore, these variations were distinctive of each cell population examined.

Studies in this area are scarce and results are often contradictory. It is difficult to compare data among various studies owing to differences in experimental designs [37]. According to some reports, an increase of the plasma level of a variety of cytokines, in particular pro-inflammatory cytokines and their soluble receptors, occurs during ageing [2, 5, 7]. In this regard, it has to be considered that the production of most cytokines is not confined to one cell type and thus it is difficult to identify the cellular sources that contribute to their plasma levels [46–48]. The technique of intracellular cytokine staining [49] offers the possibility of evaluating the contribution of different cells to the production of cytokines in heterogeneous cell populations. Using this technique, it was possible to measure intracellular cytokines and cell surface markers simultaneously to identify specific subpopulations of human leukocytes and to characterize their cytokine production [50–55].

We evaluated the cytokine pattern of adaptive immune cells (CD4+ T-cells, CD8+ T-cells and B-cells), considering that this arm of the immune system is specially targeted during ageing. As expected, some cytokines, such as IL-10 produced by T-cells declined with ageing. However, production of TNF-α and IFN-γ by CD4+ T-cells and IL-5 produced by both CD4+ and CD8+ T-cells were unaffected. Our group demonstrated that the frequencies of T cells changes during ageing with maximum frequencies at the beginning of adulthood (19–40 years) and decreasing until elderly age. This is particularly observed for CD4+ T-cells. In contrast, the frequency of CD8+ T-cells is well preserved during ageing declining only in individuals at 41–65 and 61–75 age ranges [34]. Results obtained by Alberti and coworkers [8] from the analysis of 47 European subjects at different ages indicated that the percentage of IFN-γ+-positive CD4+ T-cells significantly decreased in old and nonagenarian individuals in comparison with young subjects while the percentage of TNF-α-positive activated/memory CD4+ T-cells significantly decreased in old subjects in comparison with young ones. Differences on the methodology, age ranges and genetic background of the population studied could explain these distinct findings.

Immunosenescence studies in humans have mainly focused on the impairment of the T-cell compartment. However, changes are not limited to T-cells; B-cells have been studied as well. These cells showed a decreased production of TNF-α and IL-10 in adults and in the elderly group [12, 56, 57]. Our group has observed that frequencies of B-cells decline in Brazilian individuals of 19–40 age group and these data is compatible with the lower percentage of cytokine-positive B-cells in adulthood [34].

Although changes in T and B-cell functions were identified in healthy elderly, innate immune responses seem to be more resistant to age-associated changes [1]. NK-cells are one of the cellular mediators of innate defense and have been extensively studied in the elderly. The number and cytotoxicity activity of NK-cells are increased in healthy elderly and centenarians. Moreover, cumulative evidence in the last two decades supports the importance of NK-cell activity in maintaining good health during ageing. This is consistent with the well-preserved NK-cell cytotoxicity in centenarians fitting other criteria of healthy status such as physical fitness, independence to perform daily activities, or adequate cognitive function [18, 21, 23, 24]. Our group reported a significant increase in frequency of CD16+ IFN-γ+ NK-cells in non-infected individuals who inhabit schistosomiasis endemic areas of Brazil, and these cells are maintained at higher frequency in subjects over 70 years old. This suggests that the healthy individuals (who remain non-infected in endemic areas) are those who sustain a high frequency of IFN-γ+ NK-cells as they age [22]. In accordance with that, our results demonstrated that healthy ageing was related to a higher frequency of NK-cells that produce both a type 1 (IFN-γ) cytokine, and a counterbalancing type 2 (IL-4) cytokine.

Other innate immune cells seem to have a role in the immune-senescence process. Cellular components of the innate immune system, including neutrophils and macrophages, are the first to arrive at the site of injury. Their role is to initiate an inflammatory response, phagocyte the pathogen (in case of infection), recruit NK-cells, and facilitate the maturation and migration of dendritic cells (DCs) that regulate and determine the nature of the T-cell-mediated outcome [18]. Interestingly, frequencies of TNF-α-, IFN-gamma- and IL-4-producing neutrophils decreased from birth to adulthood and were maintained until senescence. On the other hand, frequency of TNF-α+ monocytes had a high plateau at childhood and decrease only at senescence. Of note, at this life stage, the reduced frequency of IL-10 production by adaptive immune cells was counter-balanced by an increase in the frequencies of IL-10+ cells from the innate compartment (neutrophils and monocytes).

Production of cytokines and their sources should not be evaluated alone during a complex process such as immunosenescence. The immune response is the result of several events that involves recruitment of cells, production of cytokines and chemotactic factors, establishment of immunoregulatory and proinflammatory factors. In this context, we performed a cytokine profile to assess a global picture of the immune response during the ageing process. This kind of analysis took into account the importance of the environment to an efficient immune response. This alternative strategy has been used by our group in a previous study [38], and it proved to be a useful tool to understand some of the complex immunological changes occurring during ageing.

Another important aspect of this study was the inclusion of a group of children. The childhood represents a critical period of immune development and there is a lack of data on the distributions of immunological parameters in healthy children [58]. We demonstrated that newborns had a high frequency of IL-5-producing T-cells, while neutrophils producing type 1 cytokines predominated in the innate immune compartment, a profile that remained in children. Exposure to an increasingly diverse range of antigens during the first years of life could be responsible for the environmentally driven type 1 response observed in the childhood period [59, 60]. These changes seem to promote a healthier immune profile, considering that failure in suppressing exacerbated type 2 immune responses and decreased capacity in producing type 1 cytokines during infancy are characteristic of individuals prone to develop allergic diseases [59–61].

Adolescents (11 to 20 years of age) presented an immunological profile characterized by balanced frequencies of adaptive cells able to produce both proinflammatory and regulatory cytokines. Adequate exposure to environmental antigens during infancy and the rise in sex hormones could explain the change into a robust and balanced immune profile with a predominant contribution of cytokine-producing adaptive immune cells in these individuals.

The immune system reached equilibrium in adulthood, when the cytokine profile was characterized by a balance contribution of innate and adaptive compartments to the production of both inflammatory and regulatory cytokines. Observing the overall cytokine production in individuals from the 25–51 age (Middle Aged) group, one could notice that it can be already observed a decline in cytokine production by adaptive immune cells and an expansion of the contribution of innate cells to cytokine secretion. It is widely accepted that T cells are specially targeted during immunosenescence. A number of factors have been linked to the decline in T-cell function with ageing. Age-related thymic atrophy, decreased output of naïve T-cells and the resulting memory-type oligoclonal T-cell repertoire are of particular importance. All these changes are progressive and start at different time points of adulthood [15–17, 33].

As individuals age, major immunological events such as T-cell activation and cytokine production are progressively altered. Interestingly, in all analyses performed, the elderly group presented a balanced profile between regulatory and proinflammatory cytokines, with a prominent contribution of innate immune cells to this balance. Our results support the hypothesis that healthy immunosenescence involves remodeling and adaptation to the changes started in adulthood and aggravated by ageing [32, 33].

## Conclusions

Taken together, our results showed a distinct pattern of changes in cytokine production by different leukocyte subsets during ageing. Furthermore, healthy ageing in this Brazilian population seems to involve alternative mechanisms to maintain an appropriate balance between regulatory and proinflammatory responses with well-preserved innate immunological activities compensating the impaired adaptive immune response at senescence.

The present study has limitations that should be acknowledged. First, we did not used Body Mass Index (BMI), body composition or nutritional behavior for this analysis, due to the unavailability of tools at the time of blood collection. Second, a small number of individuals was used preventing a definitive proposal of these profiles as representative of Brazilian individuals in general. One positive aspect about our cohort, however, is that the individuals are from the state of Minas Gerais, which is considered by demographic standards the most representative of the Brazilian population [62]. Therefore, despite the limitations, this study presents also important strengths. It is the first report describing changes in the profile of cytokine-producing leukocytes in Brazilian individuals within a large spectrum of age (0–85 years). These findings are an extension of another study from our group [34] that analyzed the frequency of sub-populations of leukocytes in Brazilian individuals at similar age range (0–85 years).

Data presented here may help to establish immunological reference values for future studies in Brazilian individuals at all ages. Longitudinal studies may reveal in more detail the role of innate immune cells in the elderly. Due to the progressive increase in the aged population in Brazil, these studies are critical for design and implementation of public policies aimed to optimize immune function and to improve the quality of life at advanced age.

## Additional files

Additional file 1: Figure S1. Representative scatter distribution of cytokine-producing cell subsets from the innate immunity compartment of peripheral blood. Overall distribution of cytokine + neutrophils, monocytes (CD14+) and NK-cells (CD16+) was plotted as a function of age (ranging from 0 to 85). Age ranges were established based on the overall variation rhythm observed, considering the moving mean of all cytokine+ cell subsets (continuous lines). The selected age ranges were referred as: Newborn – 0 years; Children – 6–10 years; Adolescent – 11–20 years; Adults – 21–50 years; Middle Aged – 51–60 years and Elderly – 61–85 years. Dashed rectangles were used to confine data into each established age range. (PDF 264 kb)
Additional file 2: Figure S2. Representative scatter distribution of cytokine-producing cell subsets from the adaptive immunity compartment of peripheral blood. The overall distribution of cytokine + T-cells (CD4+ and CD8+) and B-cells (CD19+) was plotted as a function of age (ranging from 0 to 85). Age ranges were established based on the overall variation rhythm observed, considering the moving mean of all cytokine + cell subsets (continuous lines). The selected age ranges were referred as: Newborn – 0 years; Children – 6–10 years; Adolescent – 11–20 years; Adults – 21–50 years; Middle Aged – 51–60 years and Elderly – 61–85 years. Dashed rectangles were used to confine data into each age range established. (PDF 333 kb)

## Abbreviations

IFN-γ: Interferon gamma
IL-10: Interleukin 10
IL-4: Interleukin 4
NK-cells: Natural killer cells
TNF-α: Tumor necrosis factor alpha
