# Supplementary material for: Lifewide profile of cytokine production by innate and adaptive immune cells from Brazilian individuals
Source: Immun Ageing. 2017 Jan 23;14:2. doi: 10.1186/s12979-017-0084-5 (PMC5260119; doi:10.1186/s12979-017-0084-5)
Supplement: Additional file 1: Figure S1. — Representative scatter distribution of cytokine-producing cell subsets from the innate immunity compartment of peripheral blood. Overall distribution of cytokine + neutrophils, monocytes (CD14+) and NK-cells (CD16+) was plotted as a function of age (ranging from 0 to 85). Age ranges were established based on the overall variation rhythm observed, considering the moving mean of all cytokine+ cell subsets (continuous lines). The selected age ranges were referred as: Newborn – 0 years; Children – 6–10 years; Adolescent – 11–20 years; Adults – 21–50 years; Middle Aged – 51–60 years and Elderly – 61–85 years. Dashed rectangles were used to confine data into each established age range. (PDF 264 kb) [file 12979_2017_84_MOESM1_ESM.pdf]

Establishment of Age Ranges Based on the Immunological Rhythm of Innate Immunity

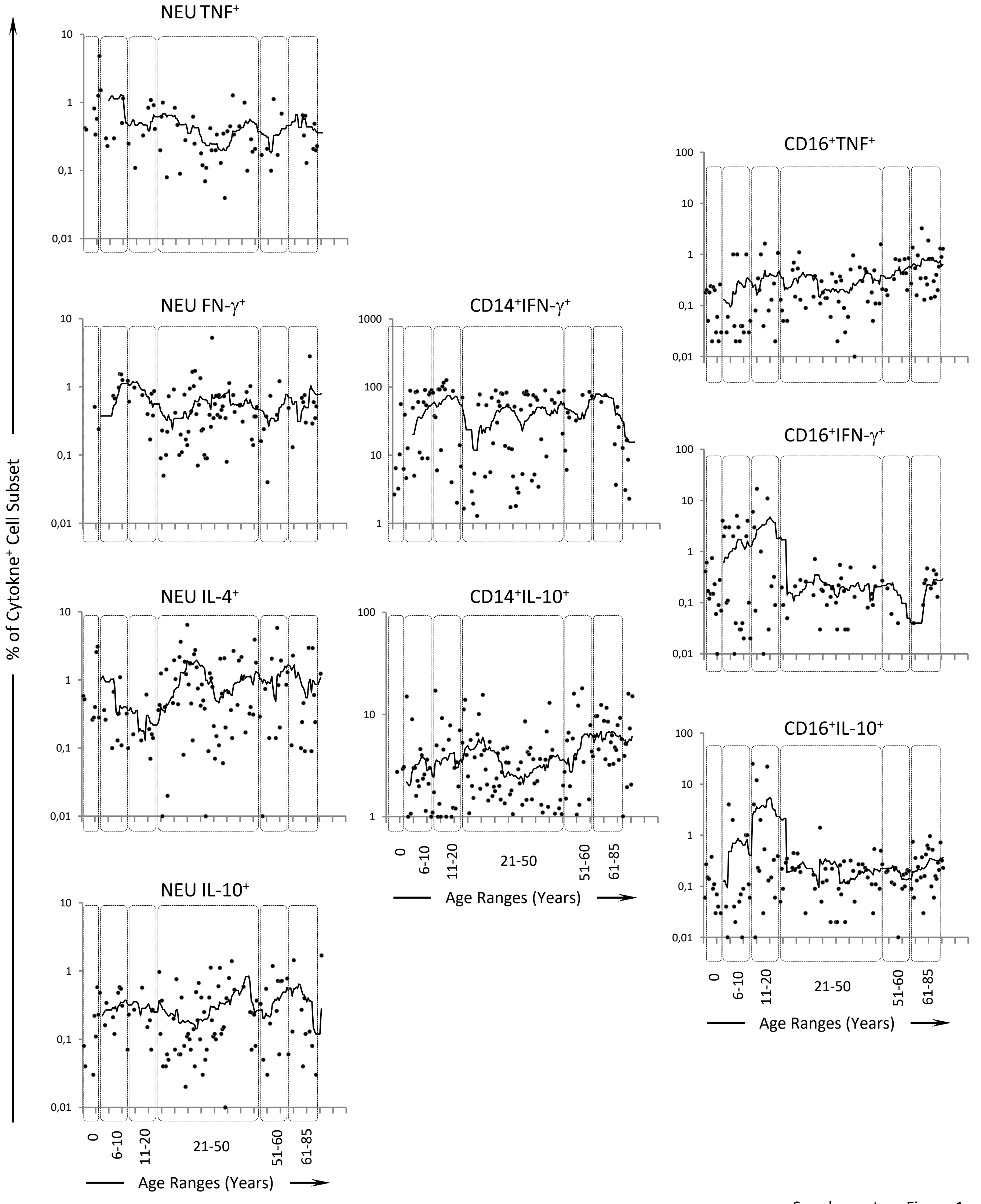

Supplementary Figure 1
