# Supplementary material for: Lifewide profile of cytokine production by innate and adaptive immune cells from Brazilian individuals
Source: Immun Ageing. 2017 Jan 23;14:2. doi: 10.1186/s12979-017-0084-5 (PMC5260119; doi:10.1186/s12979-017-0084-5)
Supplement: Additional file 2: Figure S2. — Representative scatter distribution of cytokine-producing cell subsets from the adaptive immunity compartment of peripheral blood. The overall distribution of cytokine + T-cells (CD4+ and CD8+) and B-cells (CD19+) was plotted as a function of age (ranging from 0 to 85). Age ranges were established based on the overall variation rhythm observed, considering the moving mean of all cytokine + cell subsets (continuous lines). The selected age ranges were referred as: Newborn – 0 years; Children – 6–10 years; Adolescent – 11–20 years; Adults – 21–50 years; Middle Aged – 51–60 years and Elderly – 61–85 years. Dashed rectangles were used to confine data into each age range established. (PDF 333 kb) [file 12979_2017_84_MOESM2_ESM.pdf]

Establishment of Age Ranges Based on the Immunological Rhythm of Adaptive Immunity

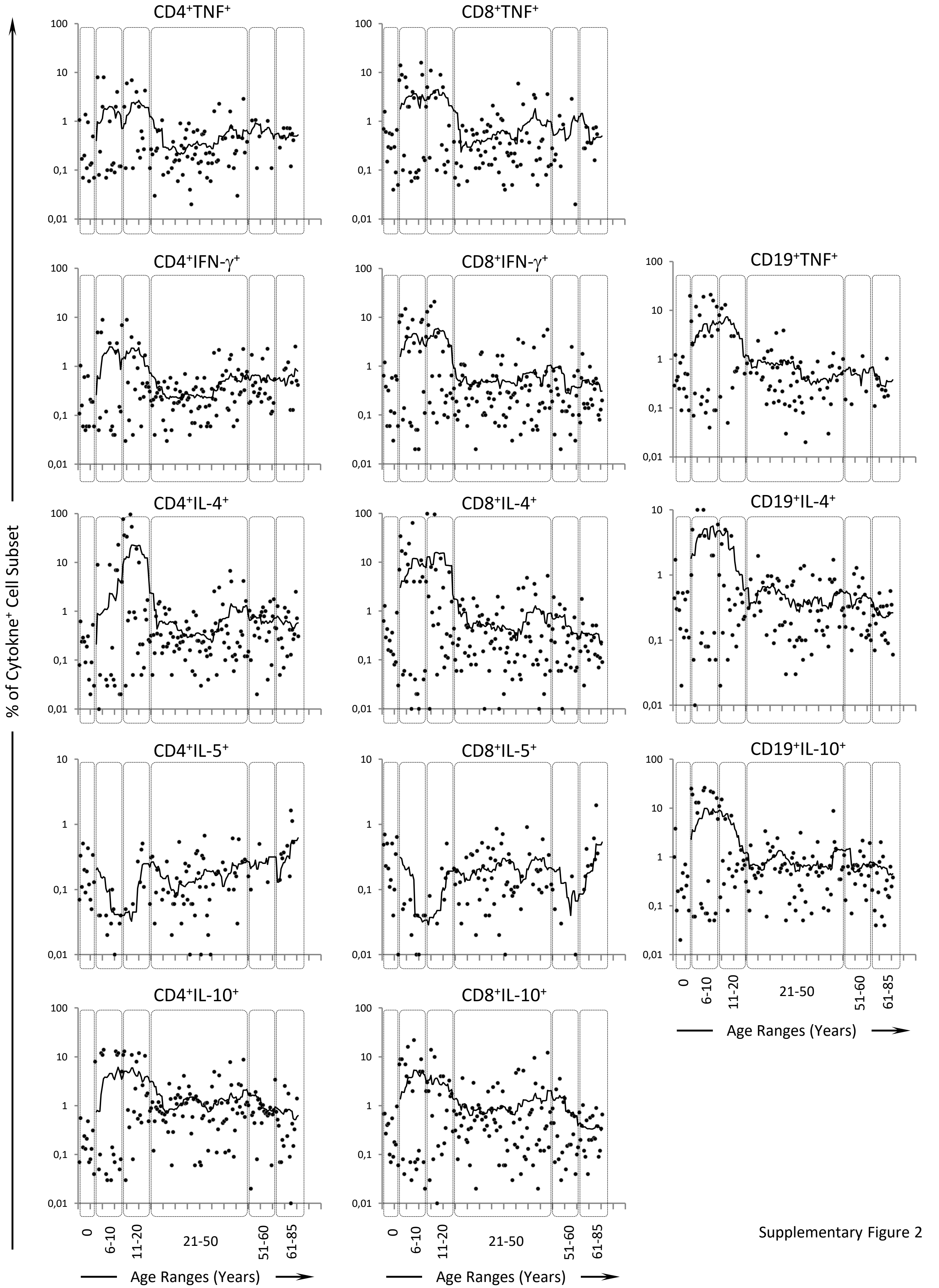

Supplementary Figure 2
